# Supplementary material for: From In Vitro to In Vivo: A Rational Flowchart for the Selection and Characterization of Candidate Probiotic Strains in Intestinal Disorders
Source: Microorganisms. 2023 Mar 30;11(4):906. doi: 10.3390/microorganisms11040906 (PMC10142325; doi:10.3390/microorganisms11040906)
Supplement: Supplementary file 1 [file microorganisms-11-00906-s001.zip › microorganisms-2279002-supplementary.pdf]

SUPPLEMENTARY DATA

Material and methods supplementary data

In vitro immunomodulation assays

For assessing immunomodulation on intestinal cells, HT-29 cells were seeded in 24-well plates (1.8 × 10^5 cells per well). Bacteria at a multiplicity of infection (MOI) of 40 or controls —either PBS glycerol, or butyrate at 1 mM—were added on the cells with recombinant TNF-α (Peprotech, London, UK) at a final concentration of 5 ng/ml. Each treatment was applied in 3 wells (technical triplicates). After 6 h of co-incubation, supernatants were recovered and stored at -80 °C until subsequent analysis. Interleukin-8 (IL-8) was later quantified in the supernatants using Human IL-8 ELISA MAX Standard Set (BioLegend, San Diego, CA, USA) according to the manufacturer's instructions. Each strain was tested 3 times from 3 independent cultures.

Immunomodulation on PBMCs was assessed on cells from five healthy donors (American male, Caucasian, aged <65 years, with a body mass index <30, nonsmoking, no drugs taken 15 days prior to sampling and negative for HIV and hepatitis A and B viruses). After thawing at 37 °C, cells were transferred into medium containing RPMI 1640 (Gibco) supplemented with 10% FBS, 1% L-glutamine, and 0.01% P/S. DNase (10 mg/mL) was added to this mix to avoid clumping. Cells were then centrifuged at 200x*g* for 15 min, counted by using trypan blue, and seeded onto 24-well plates at 1x10^6 cells/well. Bacteria at a MOI of 10 or controls —either PBS-glycerol, lipopolysaccharides (LPS) from *Escherichia coli* K-235 at 5 µg/mL (Sigma) or a mix of phorbol 12-myristate 13-acetate (PMA) and ionomycine at 2 µg/mL (eBioscience™ Cell Stimulation Cocktail, Thermofisher) — were added on cells and co-cultures were maintained for 24 h. Each treatment was tested in 3 wells (technical triplicates). After 24 h of co-incubation at 37°C in 10% CO_2, supernatants were recovered and stored at -80 °C until subsequent analysis. IL-10 and IL-12p70 were later quantified in supernatant by ELISA (Mabtech, Cincinnati, OH, USA) according to the manufacturer's instructions. Each strain was tested 3 times from 3 independent cultures.

In vitro permeability assay

For the assessment of the ability of strains to reinforce the intestinal barrier, transepithelial electrical resistance (TEER) was measured on Caco-2 cells, as previously described[22]. Briefly, Caco-2 cells were grown on Transwell® inserts (polycarbonate membrane with 0.4 µm pore size; Costar, Corning Life Science, Kennebunk, ME, USA) for about 10 days, with regular TEER measures (REMS AutoSampler, World Precision Instruments, Sarasota, FL, USA). When measures reached 2000 Ω, bacteria at MOI of 40 or the controls (either the bacteria vehicle as negative control (PBS glycerol) or an internal positive control (butyrate at 1 mM)) were co-incubated on the apical side of the cells. Three hours later, TNF-α (100 ng/mL final concentration, Peprotech) was added to the basolateral compartment. Each treatment was tested in 2 wells (technical duplicates). TEER was measured just before co-incubation and 24 h after. A ratio measuring the evolution of TEER over 24 h with the treatment, normalized by the evolution of TEER with PBS was calculated:

$$\frac{\boldsymbol{TEER\ traitement\ t24/TEER\ traitement\ t0}}{\boldsymbol{TEER\ t\acute{e}moin\ t24/TEER\ t\acute{e}moin\ t0}}.$$

Each strain was tested 3 times from 3 independent cultures.

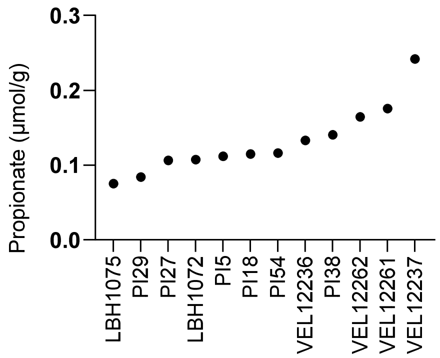

Figure S1. Propionate production in the supernatants (only the most producing strains are represented). Each strain was tested once, in technical duplicate. Quantification threshold for acetate: 0.05 mM.

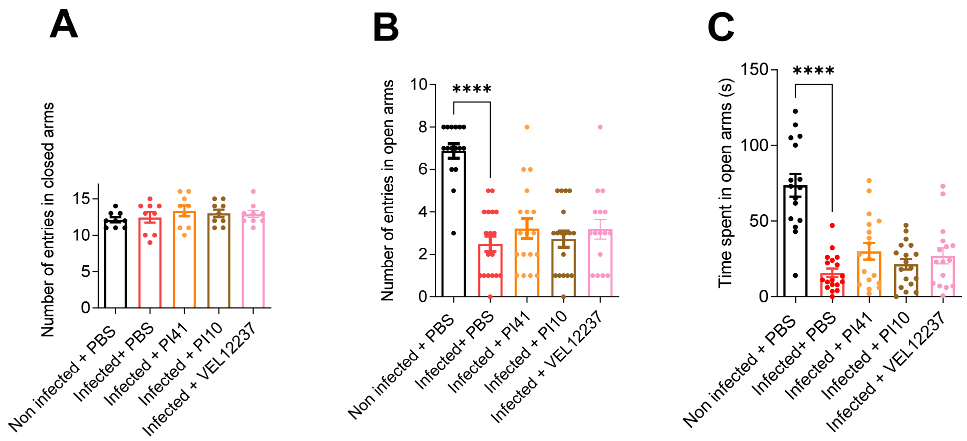

Figure S2. Effect on anxiety evaluated with Elevated Plus Maze test. A : Number of entries in the closed arms, reflecting the mobility of mice. Kruskal-Wallis non-significant. B: Number of entries in open arms as an indirect measure of anxiety. Kruskal-Wallis *p*<0.0001. C: Time spent in open arms, as an indirect measure of anxiety. Kruskal-Wallis *p*<0.0001. Infected + PBS n=17 ; Infected + PI10 n=18 ; Infected + PI41 n=18 ; Non infected + PBS n=17. Kruskal-Wallis test followed by Dunn's post-test. **** = *p* ≤ 0.0001. (mean ± SEM).
